# Compliance with the ISO 27020:2019 norm of a sample of currently available preadjusted Orthodontic bracket systems. Are the actual dimensions as expected?

**DOI:** 10.1186/s13005-021-00276-0

**Published:** 2021-07-07

**Authors:** Laura Bernés Martínez, Daniele Garcovich, Pilar España Pamplona, Milagros Adobes Adobes Martín, Alfonso Alvarado Lorenzo

**Affiliations:** 1 Department of Dentistry, Universidad Europea de Valencia, Paseo de la Alameda 7, 46010 Valencia, Spain; 2 Department of Oral Surgery, Universidad de Salamanca, Avenida Alfonso X el Sabio s/n, 37007 Salamanca, Spain

**Keywords:** Orthodontic Brackets, Slot parallelism, Slot dimension, ISO standard, Machining accuracy

## Abstract

**Background:**

Determine the exact slot dimension of a sample of a MBT prescription stainless steel conventional brackets from different manufacturers to compare the actual values with the nominal ones declared by the manufacturers and to verify the compliance with tolerance limits given by the ISO 27020:2019. Different batches from each manufacturer were evaluated to determine whether or not they are different in size. In addition, the geometry of the slot walls was assessed.

**Methods:**

360 stainless steel preadjusted orthodontic brackets of 12 different manufacturers were assessed. All brackets had a nominal slot size of 0.022 by 0.028 inches, belonged to the right upper central incisor, and were fabricated with the metal injection molding technique (MIM). For each manufacturer, three different manufacturing batches were evaluated. Brackets were coded using a single-blind design.

**Results:**

All bracket systems in the study group except one displayed a statistically significant difference with the nominal declared value, although only four of the systems did not comply with the tolerance limits established by the ISO 27020:2019. In most of the systems, the slot height was oversized when compared to the nominal one. A significant interbatch variability was found in most of the evaluated systems. Most of the brackets walls were divergent.

**Conclusions:**

The dimensional accuracy of commercially available metal brackets is not guaranteed. The respect for the norm should be enforced as well as the quality controls along the manufacturing process since orthodontic brackets are a precision medical device.

## Background

The development of the straight wire philosophy by Andrews in the '70s was a game-changing breakthrough in the field of orthodontics[1]. Despite the very many techniques launched on the market in the last decades, the vast majority of orthodontists around the world routinely use preadjusted edgewise, standard size, siamese, stainless steel brackets, conventionally ligated [2–4].

Pre-adjusted edgewise systems are systems in which every bracket bears, either in the base or in the slot, the tip, torque and in-out information tailored to the ideal values for each tooth. From a theoretical standpoint, inserting a full-size arch in the slot should allow the full expression of the preadjusted values, helping to reach the ideal alignment of the dentition [5–7]. Nevertheless, in a clinical setting, the orthodontists still need, in most of the cases to bend wires to reach the ideal alignment and intercuspation. Torque expression is regarded as crucial [5] therefore most of the authors have focused on this feature, especially regarding anterior teeth. The correct position of the incisors is pivotal to get a good sagittal occlusion and has a relevant impact on the arch length and on the incisor display from an aesthetic standpoint [8]. Torque expression could be altered by different factors such as a peculiar dental anatomy or errors in brackets positioning [8, 9], nevertheless, the key factor for the full expression of torque is the information inbuilt in the bracket itself [8, 9].

To reach the treatment goals by means of a straight-wire appliance is fundamental to rely on brackets manufactured with accuracy, with reliable preadjusted values of torque, tip and in-out. To express all inbuilt information, the slot geometry has to allow the full contact of a full-size wire with the slot´s walls. The discrepancy between the declared prescription values and the real ones will result in an incorrect transmission of the information that the slot should deliver to the tooth, resulting in its incorrect three-dimensional positioning [5, 9, 10].

The grounding assumption of the straight-wire technique is therefore the correct bracket manufacturing to deliver the required force system and achieve the planned three-dimensional position of the dentition. Quality norms have been established to indicate to the manufactures the required standards [6, 11]. The ISO 27020:2019 norm, approved by the European Committee for Normalization, refers to brackets and tubes used in orthodontics and is the reference norm in the European Union. The norm specifies the dimensional tolerance limits or the standardised measure of the maximum dimensional difference between the declared dimensions and the actual ones [11].

The aim of our study was to determine the exact slot dimension of a sample of McLaughlin, Bennet, and Trevisi (MBT) prescription stainless steel conventional brackets from different manufacturers and compare the actual values with the nominal dimension. Moreover, the compliance with the tolerance limits given by the ISO 27020:2019 norm along the different batches from the same manufacturer was explored as well as the geometry of the slot walls.

## Methods

### Sample

A pool of 360, stainless steel preadjusted orthodontic brackets of 12 different manufacturers was assessed. All brackets had a nominal slot size of 0.022 by 0.028 inches, belonged to the right upper central incisor, and were fabricated with the metal injection molding technique (MIM). The sample involved the following brackets systems: SYNTHESIS® (Ormco, Orange, California, USA); NU-EDGE® (TP Orthodontics, La Porte, Indiana, USA); DISCOVERY SMART® (Dentaurum, Ispringen, Germany); FLI TWIN® (RMO, Denver, Colorado, USA); MINI SPRINT® (Forestadent, Phorzheim, Germany); OMNIARCH+® (Dentsply GAC, Bohemia, New York, USA); SCAPE® (Ah Kim Pech Corporation, Ciudad de Mexico, Mexico); MINI-TWIN® (Lancer Orthodontics, Carlsbad, California, USA); JAZZ® (Modern Orthodontics, Ludhiana, India); MINI MASTER® (American Orthodontics, Sheboygan, Wisconsin, USA); MINIPREVAIL® (G&H Orthodontics, Franklin, Indiana, USA) and VICTORY® (3 M, Monrovia, California, USA). All systems were preadjusted according to the MBT technique values. For every system, 30 brackets belonging to three different batches were assessed, 10 brackets per batch. All batches were fabricated between January 2019 and March 2020. A sample size calculation was undertaken using the software Raosoft® Version 5.0 (Raosoft, Inc., 6645 NE Windermere Road, Seattle, WA 98,115). It was determined that a sample size of 9 was enough to detect a difference of 0.003 mm, assuming a standard deviation of 0.002 mm with a 90 % power and significance level of p < .05. It should be underlined that the ISO 27020:2019 norm testing protocol requires to test 6 brackets for every batch. Once the brackets were received, they were randomly coded by a member of the research group (MAM), and stored in such a way that the other two team members, who performed the actual measurements were not aware of the system they were assessing neither of the batch number, using a single-blind design.

### Measurement protocol

The brackets dimensions were registered by means of a digital stereo microscope LEICA® DMS 1000 (Leica Microsystems gmbh, Wetzlar, Germany). The Images were captured and processed using the LAS V4.5® CORE software. The same software was used to obtain the desired measurements. The brackets were positioned on the stereo microscope tray using rope wax with the slots oriented vertically, with the distal side up, so that the line of view on the stereo microscope was parallel to the slot main axis. The light was adjusted until a sharp, well-focused image was obtained on the screen and digitally captured.

To assess the intraoperator reliability, a set of 15 randomly selected brackets belonging to the study group, were measured twice at a two-week interval by the principal investigator (LB). The interoperator reliability was assessed by measuring the same set of brackets by a second operator (DG).

**Fig. 1:**
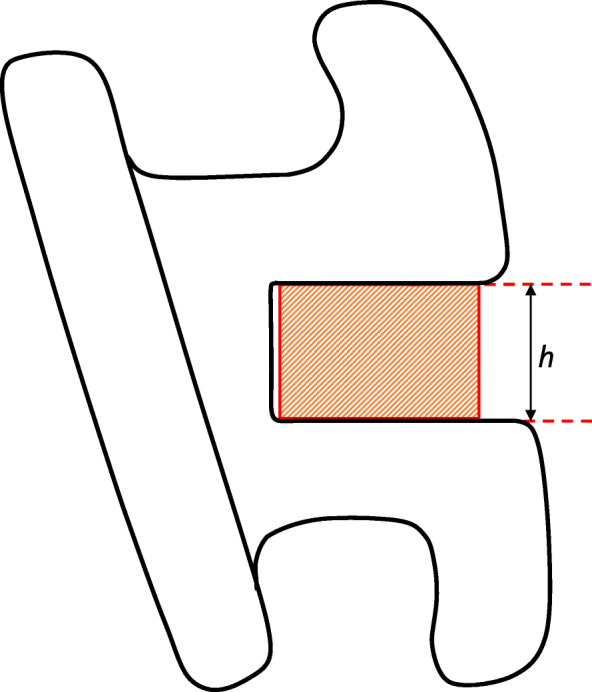
Determination of the bracket slot height according to ISO 27020:2019 protocol. (h) is the maximum occluso-gingival dimension of the hypothetic rectangle that fills completely the bracket’s slot

The ISO 27020:2019 describes the testing protocol to be used to assess the slot´s dimensions and the current study adopted the method described by the norm. The slot height is defined by the norm as the maximum occluso-gingival dimension of the hypothetic rectangle that fills completely the bracket´s slot, measured perpendicular to the slot mesiodistal main axis (Fig. 1). The nominal bracket height is usually expressed by the manufacturer in inches, but the norm adopts the metric system, so the measurements were registered in millimetres.

**Fig. 2:**
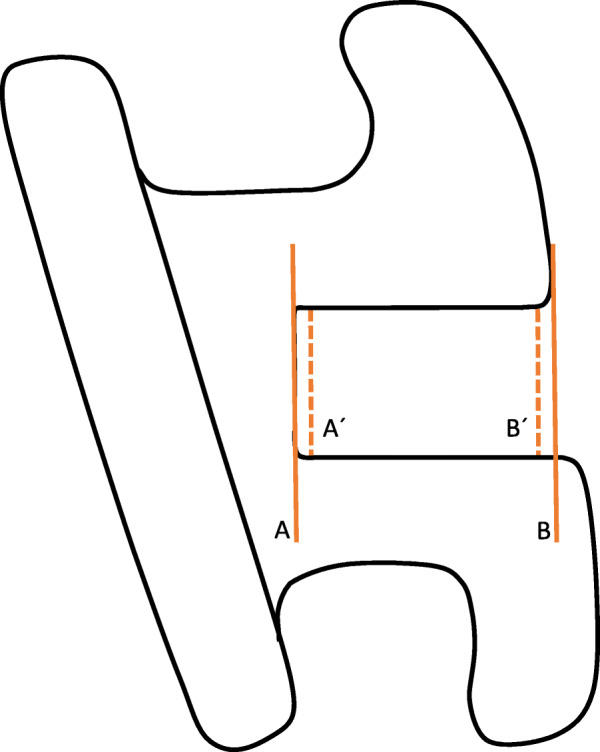
Assessment of the bracket slot geometry. (**A**) line tangent to the slot bottom. (**B**) Line parallel to (**A**) line and tangent to the outermost part of the slot. Internal height (**A´**) and external bracket slot height (**B´**) have been calculated over two lines parallel to lines (**A**) and (**B**) at a 100 μm distance from lines (**A**) and (**B**)

To determine the bracket slot geometry, the slot height was measured at two different points. The internal height (Height A´) was calculated at 100 µm from the slot deepest point to prevent the bias due to the roundness of the slot angles, and the external height (Height B’) at 100 μm from the outer border of the slot, when the upper and lower borders were different in length, the shorter one was taken as a reference (Fig. 2). The 100 μm distance to the deepest point of the slot is used in most of the articles assessing the slot height [12, 13]. The difference between the external and internal height (Height B’-Height A´) was used to determine the slot geometry. The walls were considered parallel if the difference value was 0, divergent if positive, and convergent if negative. The mean internal and external heights were then compared in each bracket system to highlight any discrepancy.

### Statistical analysis

The dimensional data were collected on an Excel datasheet (Microsoft Office for Mac 2011 package). The statistical analyses of the data distribution and statistical significance were performed using a SPSS software (version 25.0; IBM Corp., Armonk, NY, USA). The method error, and the intra and interoperator reliability were estimated by mean of the Dalberg formula and the Intraclass Correlation Coefficient (ICC). A confidence interval of 95 % (CI 95 %) between means was used. After assessing the normality of data distribution by means of a Kolmogorov-Smirnov test, a non-parametric Wilcoxon test was used to compare the actual with the nominal values. To compare the means of the values of the different batches of the same system, a multivariate ANOVA test was used in the cases where the distribution was considered normal and a Kruskal-Wallis test in case it was not normal. To evaluate the slot geometry, the mean internal and external height were compared through a parametric t-test or a non-parametric Wilcoxon test depending on the normality of data distribution in each system.

## Results

### Intra and interoperator reliability

The Dahlberg error and the ICC for repeated measurements were 0.00 mm and 0.992 respectively for intraoperator reliability while they were 0.01 mm and 0.0980 for interoperator reliability, being the method error low and thereby describing a high intra-operator reliability and suggesting a high repeatability of the measurements.

### Slot height

The nominal slot height declared by the manufacturers is 0.022 inches, which is equivalent to 0.559 millimetres. According to the ISO 27020:2019, the industrial tolerance for slot height is ± 0,01mm, being the acceptable range between 0.549 and 0.569 mm.

The descriptive statistics for the bracket slot heights are shown in Table 1. All systems in the study group except for MINIPREVAIL® (*p* = .078), displayed a statistically significant difference between the actual and nominal slot height as declared by the manufacturer. Moreover, four of the systems presented a mean slot height out of the tolerance limit established by the ISO 27020:2019 norm (In boldface in Table 1).

**Table 1:** Bracket slot height in the analysed systems. In boldface the systems with a mean slot height out of ISO 27020:2019 tolerance limit

Bracket system	N	Mean (± SD)	Min	Max	MD	Z_W_	*p*
SYNTHESIS	30	0.556(± 0.004)	0.554	0.559	-0.003	-4.616	< 0.001***
NU-EDGE	**30**	**0.574(± 0.010)**	**0.559**	**0.591**	**0.015**	**-4.784**	< 0.001***
DISCOVERY	**30**	**0.570(± 0.010)**	**0.558**	**0.590**	**0.011**	**-4.476**	< 0.001***
FLI TWIN	30	0.566(± 0.008)	0.558	0.590	0.007	-4.560	< 0.001***
MINI SPRINT	30	0.567(± 0.005)	0.558	0.577	0.008	-4.705	< 0.001***
OMNIARCH +	30	0.560(± 0.002)	0.555	0.567	0.001	-2.575	0.010**
SCAPE	30	0.562(± 0.006)	0.558	0.571	0.003	-3.564	< 0.001***
MINI-TWIN	30	0.554(± 0.005)	0.542	0.562	-0.005	-3.924	< 0.001***
JAZZ	**30**	**0.548(± 0.009)**	**0.528**	**0.560**	**-0.011**	**-4.456**	< 0.001***
MINI MASTER	**30**	**0.548(± 0.005)**	**0.540**	**0.559**	**-0.011**	**-4.766**	< 0.001***
MINIPREVAIL	30	0.560(± 0.004)	0.552	0.570	0.001	-1.763	0.078
VICTORY	30	0.560(± 0.001)	0.558	0.563	0.001	-3.953	< 0.001***

*SD* Standard deviation; *Min* Minimum slot height; *Max* Maximum slot height; *MD* Mean difference between nominal and actual values; *Z*_W_ Wilcoxon standardized rank; *Wilcoxon test* *p ≤ .05; **p < .01; ***p < .001

**Fig. 3:**
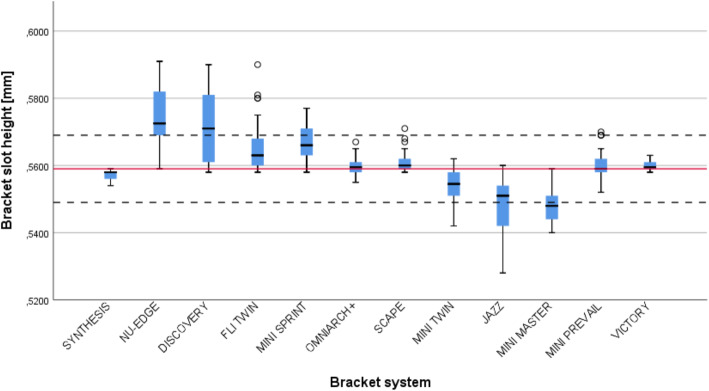
Box plot showing the median and interquartile ranges for the bracket slot height. Continuous red line shows the nominal value of bracket slot height. Dashed lines represent the upper and lower tolerances limits of the ISO 27020:2019 norm

In most of the systems, the slot height was oversized when compared to the nominal one, but in four systems, JAZZ®, MINI MASTER®, SYNTHESIS® and MINI-TWIN®, it was undersized. The first two systems were undersized and out of the tolerance limits, while the last two were within the limits, as shown in Table 1; Fig. 3.

The smallest slot height of the whole pool was displayed by the JAZZ® system with 0.528mm, while the largest was in the system NU-EDGE® with 0.591mm. At a global level, it can be stated that in the pool the bracket slots were undersized till 5.54 % or oversized up to 5.73 %, compared to the nominal value. (Table 1; Fig. 3).

The distribution of the slot height value in every system is displayed in Fig. 3. The Boxplot shows the median and interquartile ranges for the bracket slot height. NU-EDGE®, DISCOVERY® and JAZZ® were the systems showing the highest data dispersion, being a high number of slots out of the tolerance limits and oversized in the first two systems and undersized in the last one.

On the contrary, OMNIARCH+®, SCAPE®, VICTORY®, SYNTHESIS® and MINIPREVAIL® were the systems where slot height displayed the lowest variability, being the actual values close to the nominal ones and into the tolerance limits.

### Interbatch variability

**Fig. 4:**
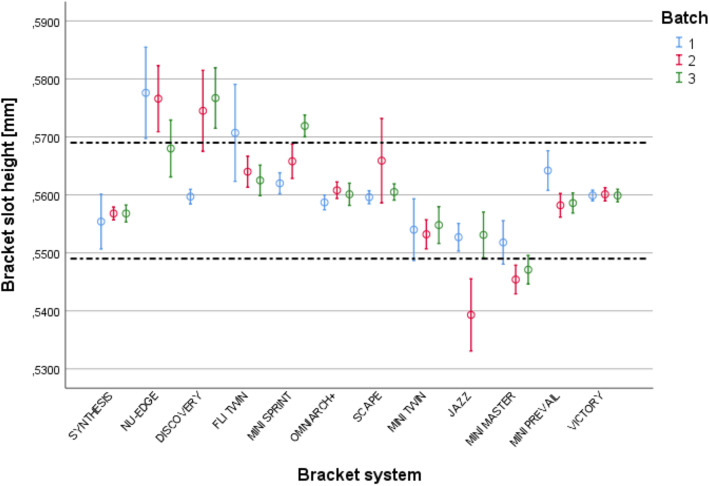
Slot height in the difference batches (Interbatch variability); the Confidence interval (CI) in mm; dotted lines represent the upper and lower tolerance limits of the ISO 27020:2010

As highlighted in Table 2, in half of the analysed systems, the differences in slot height were statistically different among the three batches. As displayed in Fig. 4, DISCOVERY®, MINI SPRINT® and JAZZ® presented the highest interbatch variability. The first of these systems presented slots out of the tolerance limits in all analysed batches. VICTORY®, SYNTHESIS®, MINI-TWIN®, OMNIARCH+® and MINIPREVAIL® were the systems characterized by a low interbatch variability and the only systems whose brackets were all within the tolerance limits established by the norm in the three batches. VICTORY® and OMNIARCH+® were the systems with the lowest interbatch variability. In seven systems, the brackets of at least one batch were out of the tolerance limits.

**Table 2:** Slot height in the different batches for each system (interbatch variability of slot height)

Bracket system	Batch 1		Batch 2		Batch 3		
	N	Mean (± SD)	N	Mean (± SD)	N	Mean (± SD)	*p*
SYNTHESIS NU-EDGE DISCOVERY FLI TWIN MINI SPRINT OMNIARCH+ SCAPE MINI-TWIN JAZZ MINI MASTER MINIPREVAIL VICTORY	10 10 10 10 10 10 10 10 10 10 10 10	0.555 (± 0.007) 0.578 (± 0.011) 0.560 (± 0.002) 0.571 (± 0.012) 0.562 (± 0.003) 0.559 (± 0.002) 0.560 (± 0.002) 0.554 (± 0.007) 0.553 (± 0.003) 0.552 (± 0.005) 0.564 (± 0.005) 0.600 (± 0.001)	10 10 10 10 10 10 10 10 10 10 10 10	0.557 (± 0.002) 0.577 (± 0.008) 0.575 (± 0.010) 0.564 (± 0.004) 0.566 (± 0.004) 0.561 (± 0.002) 0.566 (± 0.010) 0.553 (± 0.004) 0.539 (± 0.009) 0.545 (± 0.003) 0.558 (± 0.003) 0.560 (± 0.002)	10 10 10 10 10 10 10 10 10 10 10 10	0.557 (± 0.002) 0.568 (± 0.007) 0.577 (± 0.007) 0.563 (± 0.004) 0.572 (± 0.003) 0.560 (± 0.003) 0.561 (± 0.002) 0.555 (± 0.004) 0.553 (± 0.006) 0.547 (± 0.003) 0.559 (± 0.002) 0.600 (± 0.002)	0.901 0.041* < 0.001*** 0.401 < 0.001*** 0.082 0.126 0.516 < 0.001*** 0.010* 0.012* 0.965

*SD* Standard deviation; A multivariate ANOVA was performed for NU-EDGE®. DISCOVERY®. MINI SPRINT® and JAZZ® a Kruskal-Wallis test in others systems; *p ≤ .05; **p < .01; ***p < .001

### Slot Geometry

In terms of slot geometry, in only two systems, DISCOVERY® and JAZZ®, the difference between the mean internal and external slot height was not statistically significant. The upper and lower walls can be considered parallel in these systems. As highlighted in Table 3, most of the systems presented divergent walls.

**Table 3:** External and internal slot height in the different bracket systems and their differences

Bracket series	External height	Internal height	Difference External-Internal Height	
	Mean (± SD)	Mean (± SD)	Mean(± SD)	*p*
SYNTHESIS NU-EDGE DISCOVERY FLI TWIN MINI SPRINT OMNIARCH+ SCAPE MINI-TWIN JAZZ MINI MASTER MINIPREVAIL VICTORY	0.562 (± 0.006) 0.609 (± 0.016) 0.572 (± 0.012) 0.574 (± 0.011) 0.571 (± 0.007) 0.561 (± 0.005) 0.592 (± 0.013) 0.559 (± 0.008) 0.550 (± 0.010) 0.555 (± 0.005) 0.564 (± 0.007) 0.581 (± 0.030)	0.556 (± 0.004) 0.575 ± 0.010) 0.573 ± 0.012) 0.567 ± 0.008) 0.567 ± 0.005) 0.568 ± 0.006) 0.563 ± 0.008) 0.556 ± 0.006) 0.552 ± 0.010) 0.548 ± 0.005) 0.561 ± 0.004) 0.565 ± 0.012)	0.006 (± 0.005) 0.034 (± 0.014) -0.010 (± 0.008) 0.007 (± 0.005) 0.004 (± 0.004) -0.007 (± 0.008) 0.029 (± 0.011) 0.003 (± 0.006) -0.002 (± 0.007) 0.007 (± 0.004) 0.003 (± 0.005) 0.016 (± 0.022)	< 0.001*** < 0.001*** 0.656 < 0.001*** < 0.001*** < 0.001*** < 0.001*** 0.006** 0.067 < 0.001*** 0.006** < 0.001***

*SD* Standard deviation; NU-EDGE®. MINI SPRINT® and MINI MASTER® were assessed with a Student t-test while a Wilcoxon test in the other systems; *p ≤ .05; **p < .01; ***p < .001

**Fig. 5:**
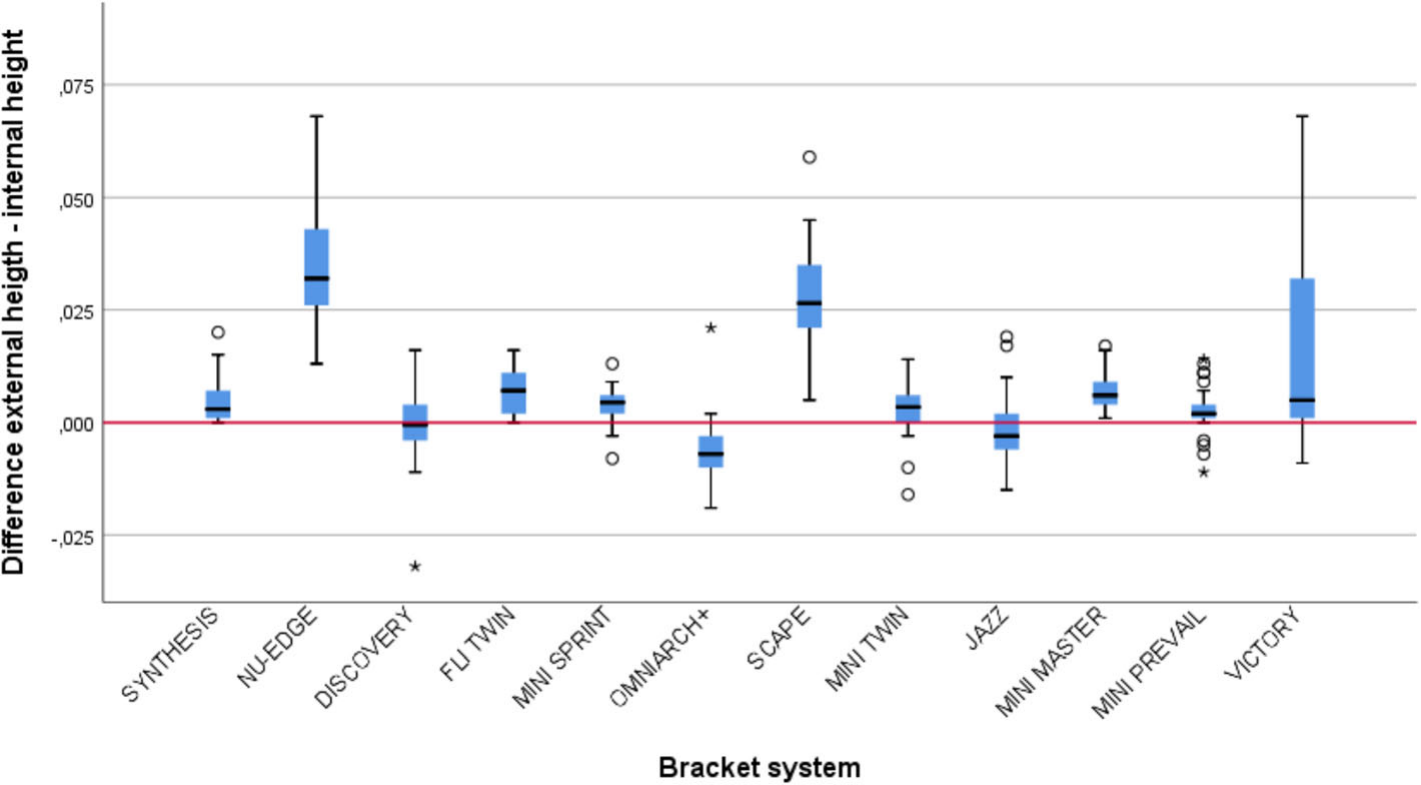
Box plot showing the median and interquartile ranges for the difference between internal and external slot heights

Figure 5 highlights how despite of being the walls generally divergent along the different systems in NU-EDGE®, SCAPE® and VICTORY® slots had the highest value of divergence and a higher value dispersion. Only in the case of OMNIARCH+® slot walls were convergent. The main results about slot height, interbatch variability and slot geometry are presented in Table 4.

**Table 4:** Slot height, Slot Geometry and Interbach variability in the different bracket systems

	Slot Height					Batch 1	Batch 2	Batch 3	
Bracket system	N	Mean (± SD)	*p*	Slot Geometry	N/ batch	Mean (± SD)	Mean (± SD)	Mean (± SD)	*p*
SYNTHESIS	30	0.556(± 0.004)	< 0.001***	<	10	0.555 (± 0.007)	0.557 (± 0.002)	0.557 (± 0.002)	0.901
NU-EDGE	**30**	**0.574(± 0.010)**	< 0.001***	<	10	0.578 (± 0.011)	0.577 (± 0.008)	0.568 (± 0.007)	0.041*
DISCOVERY	**30**	**0.570(± 0.010)**	< 0.001***	=	10	0.560 (± 0.002)	0.575 (± 0.010)	0.577 (± 0.007)	< 0.001***
FLI TWIN	30	0.566(± 0.008)	< 0.001***	<	10	0.571 (± 0.012)	0.564 (± 0.004)	0.563 (± 0.004)	0.401
MINI SPRINT	30	0.567(± 0.005)	< 0.001***	<	10	0.562 (± 0.003)	0.566 (± 0.004)	0.572 (± 0.003)	< 0.001***
OMNIARCH +	30	0.560(± 0.002)	0.010**	>	10	0.559 (± 0.002)	0.561 (± 0.002)	0.560 (± 0.003)	0.082
SCAPE	30	0.562(± 0.006)	< 0.001***	<	10	0.560 (± 0.002)	0.566 (± 0.010)	0.561 (± 0.002)	0.126
MINI-TWIN	30	0.554(± 0.005)	< 0.001***	<	10	0.554 (± 0.007)	0.553 (± 0.004)	0.555 (± 0.004)	0.516
JAZZ	**30**	**0.548(± 0.009)**	< 0.001***	=	10	0.553 (± 0.003)	0.539 (± 0.009)	0.553 (± 0.006)	< 0.001***
MINI MASTER	**30**	**0.548(± 0.005)**	< 0.001***	<	10	0.552 (± 0.005)	0.545 (± 0.003)	0.547 (± 0.003)	0.010*
MINIPREVAIL	30	0.560(± 0.004)	0.078	<	10	0.564 (± 0.005)	0.558 (± 0.003)	0.559 (± 0.002)	0.012*
VICTORY	30	0.560(± 0.001)	< 0.001***	<	10	0.600 (± 0.001)	0.560 (± 0.002)	0.600 (± 0.002)	0.965

*N* number of brackets assessed; *SD* Standard deviation; the difference between nominal and actual values was assessed through a *Wilcoxon test* *p ≤ .05; **p < .01; ***p < .001; <: divergent slot geometry; > convergent slot geometry; =: parallel slot walls; N/batch: number of brackets assessed per batch; the difference between the different batches was assessed through A multivariate ANOVA for NU-EDGE®. DISCOVERY®. MINI SPRINT® and JAZZ® a Kruskal-Wallis test in others systems; *p ≤ .05; **p < .01; ***p < .001

## Discussion

To the best of our knowledge, this is the first study to assess the slot dimensions of contemporary stainless steel brackets according to ISO 27020:2019 norm, that establishes tolerance limits for the dimension of the bracket´s slot and the inbuilt torque tip and in-out. A previous study assessed the bracket dimension according to the DIN 13971-2 norm. DIN norm despite using a similar testing protocol, admits a larger tolerance range[9]. Moreover, our study is the first one to assess the interbatch variability of the same bracket systems.

Nowadays, the most prevalent manufacturing process for brackets body is MIM, while investment casting and machining or milling are less prevalent due to their longer production cycle and lower cost-effectiveness [14]. Some bracket systems use a combination of MIM for bracket´s body manufacturing and machining or milling for the brackets slot. Unfortunately, bracket manufacturing processes are mostly proprietary and the manufactures are sharing very few information about it.

In a recent paper Jae-Sun Park et al. [15] compared the size of the slot and the parallelism of the walls of metal brackets manufactured through Metal Injection Molding (MIM) and milling through a Computerized Numerical Control (CNC) machine. The CNC software should control the process and it is theoretically designed to produce brackets in large quantities with high quality and a better dimensional accuracy when compared to other manufacturing processes as casting or injection molding. The authors found that the entire bracket sample had oversized slots and only one of the seven evaluated systems presented parallel walls, being the others divergent. They could not conclude that milling through CNC manufacturing method was more accurate than the MIM.

In the last years, 3D metal printing has appeared as an emerging technology with the potential to streamline bracket production for personalized and precision orthodontics. This technique is an additive process of manufacturing in which layers of material are added by computer control to produce a finished product. Metal printing can be performed with both stainless steel and titanium alloys, both materials commonly used in conventional orthodontics brackets [16]. Jackson (2017) compared the dimensional accuracy of novel one-piece 3D metal printed orthodontic brackets and two conventionally manufactured brackets (Damon® and Ti-Orthos®). The authors found that the mean slot height of the 3D printed slot was the closest to the nominal value (0.022 inch.), but had the largest standard deviation. The Damon® and Ti-Orthos® brackets presented significantly smaller standard deviations. Statistically significant differences were also found between the right and left slot height, probably due to the orientation of the bracket during the printing process [17]. Future studies are needed to explore the surface finishing and the dimensional accuracy of 3D printed brackets.

All analysed systems in the current study were manufactured by Metal Injection Molding (MIM) technique. MINI-TWIN® and MINI MASTER® were the only systems who combined the MIM technology for the bracket´s body manufacturing and the milling of the slot through a diamond blade. Although MIM is the most cost-effective technology for bracket manufacturing [18], it implies the use of a 18 to 20 % oversized mold to compensate the shrinkage after sintering. The shrinkage could vary depending on a large number of factors (alloy, powder type, de-binding method, sintering heat rate, sintering hold time) and affect the actual dimensions [18].

### Slot height

Almost all previous studies pointed out that there is a significant difference between the actual and nominal values of slot height, being the bracket slot oversized in most of the cases. According to our results, the slot can be 5.73 % higher than the nominal value, a value higher than the one presented by Major et al. in self-ligating brackets [19], but smaller than the one of Lefevbre et al. who found slots oversized up to 10 % in a sample of edgewise brackets [13]. Cash et al. [10] and Joch and Pichelmayer [9], found oversized slots in 100 % of the brackets assessed. Their study samples were smaller than ours and their results were published more than a decade ago, being the better performance in our sample probably related to the evolution in the manufacturing process over the last decade. Cash et al. [10] highlighted how the slot could be oversized between 2.26 and 24 % of the declared values, while Joch and Pichelmayer [9] reported an oversize range between 1 and 7 %, being their findings similar to ours. These authors referred in their investigation to the German DIN 13971-2 norm and concluded that despite the general oversized values found in the slot height, all brackets tested were within the tolerance limits reported by the norm. The DIN 13971-2 norm considers the tolerance limit as ± 0.04mm, while the limit is ± 0.01mm in the case of the ISO 27020:2019.

Kusy et al. [20], Awasthi et al. [21] and Brown et al.[12], in agreement with our findings, reported that in the majority of the systems the slot was oversized but highlighted how in some cases it could be undersized too but just in a smaller percentage of cases. They, respectively, found undersized slots in 15 %, 22.3 %, and 36 % of the analysed pool. Kusy [20] and Awasthi [21] studied a sample smaller than ours, while Brown [12] who used a similar sample size, reported a result very similar to the 33.3 % found in our study.

According to our findings (Table 1), Cash et al. [10], Brown et al. [12] and Arreghini et al. [22] also found oversized slots in a sample of VICTORY® bracket system. Brown et al. [12] reported that in a sample of MINI MASTER® brackets, the slot height was undersized when compared to the nominal value declared by the manufacturer being this finding consistent with ours.

Although according to the norm, undersized slots within the tolerance limits are considered acceptable, from a practical standpoint, they could impede the correct seating of a full size wire that has a nominal cross section that is equal to the nominal slot size [21]. In a clinical setting, the full size arch can seat also in the case of undersized slots, taking into account that contemporary archwires are usually undersized when compared to their nominal value [23]. The high prevalence of oversize slots can be partly due to a failure in calculating the actual shrinkage after sintering or by a lack of control in the final polishing phase. Moreover, quality control using gauges detects and discards easier undersized brackets than the oversized ones.

### Interbatch variability

This is the first study comparing brackets accuracy in different batches, therefore we can not contrast our results with the ones of other researchers. Many factors along the manufacturing process can induce dimensional imprecisions and, if not controlled, result in interbatch differences. The homogeneity within a batch and the consistency between batches are the final goals of process validation activities. A validated process is reasonably protected against sources of variability that could affect the production output [24]. The significant variability among different batches highlighted in the study in 6 out of 12 systems can be due to a poor process validation or the lack of an effective quality check.

### Slot Geometry

Slot geometry is a key factor in ensuring a correct bracket-to-arch contact and allow the full expression of the inbuilt prescription. Slot´s upper and lower walls have to be as parallel as possible, perpendicular to the slot bottom, smooth and without irregularities or impurities [12]. In accordance with the results of the present study, most of the authors pointed out the lack of parallelism in the slot walls. Lefebvre et al. [13] and Youngran Lee et al. [25] found in their samples mostly divergent walls, 84–85 and 100 %, respectively. Cash et al. [10] found parallel walls only in three of the eleven systems included in their study, with most of the systems presenting converging walls. According to our results, Cash et al. and Brown et al. found divergent walls in the VICTORY® and NU-EDGE® systems [10, 12]. Jae-Sun Park et al. [15] found divergent walls in seven out of the eight bracket systems assessed being the wall parallel in only one system. In their sample MIM and Milled brackets were compared and neither of the manufacturing processes proved to be able to ensure a correct geometry.

### Clinical Implications

The effective expression of the information inbuilt in the bracket´s slot relies on the proper contact of a full size wire with the slot’s walls. The torque expression is especially sensitive to the eventual play between the wire and the bracket´s slot. The imprecisions in the manufacturing process inevitably affect the play between the archwire and the slot and, therefore, the torque expression capacity of the appliance [6, 8, 9, 26]. It is pivotal to know if the appliances provided by the manufacturer respect the nominal declared values. Oversized brackets are responsible for a suboptimal torque expression or torque loss depending on the employed orthodontic mechanic. Siatkowski reported an unexpected torque loss during *en masse* incisor retraction due to slot-wire play [27]. Alexander highlighted how every 0.001 inch of play between the archwire and the slot is responsible for a torque loss of about 5º [12], forcing the orthodontics to insert manually an extra torque on the main wire or to use other torque reinforcing techniques.

Gioka and Eliades (2004) suggested that the clinicians could use different strategies to reduce the material induced torque loss as: choosing increased torque prescriptions; insert large cross-section stainless steel archwire (.021x.025 inch.); use stainless-steel ligature rather than elastomeric ones [28].

The results of the present study suggest that in most of the systems, due to a dimensional inaccuracy or to a shape distortion, the slot-arch play is not predictable. From a clinical standpoint, it could be therefore difficult to reach treatment goals without compensating through manual bending, the poor accuracy of the bracket slot. The manufacturing inaccuracy is indeed a key factor in decreasing the clinical performance of the straight wire system [21, 29]. Moreover, in the light of our results, it seems of limited value to modify the brackets prescription by a few degrees in order to reach different clinical results. The orthodontic community should not only focus in determining the right prescription to reach the treatment goals but also ask for dimensionally accurate brackets, that allow to deliver the right prescription to their patients.

### Strengths and limitations

The low method error, quantified by the inter and intraoperator reliability, the large number of systems involved, the interbatch assessment, and the single blind design adopted, can be considered strengths of the current study.

A number of limitations of this study should also be acknowledged. We did not include stainless steel brackets manufactured by procedures different than MIM or different types of brackets as the self-ligating ones. Ceramic brackets were not included in the current study either. All measurements were performed from the distal side not assessing the mesial one, although all previous studies analysed only one side [10, 12, 13, 22, 23], this protocol can hinder the slot dimensional asymmetry.

## Conclusions

Within the limitations of the current study, it can be stated that the quality standards of the ISO 27020:2019 are not respected in the vast majority of the analysed bracket systems. Adherence to the recommended standards and quality controls must be enforced throughout the manufacturing process, as orthodontic brackets are a precision medical device. Among the analysed systems, only one, displayed an actual slot height not significantly different from the nominal one from a statistical standpoint. Four of the analysed systems presented a slot height mean value out of the tolerance limits of the ISO 27020:2019. Only four systems presented a low interbatch variability with all brackets of the sample respecting the tolerance limits established by the norm. Most of the systems presented divergent walls.

## Data Availability

All data generated or analysed during this study are included in this published article.

## References

[1] Andrews LF (1976). The straight-wire appliance. Explained and compared. J Clin Orthod.

[2] Alavi S, Tajmirriahi F (2016). Assessment of dimensional accuracy of preadjusted metal injection molding orthodontic brackets. Dent Res J (Isfahan).

[3] Banks P, Elton V, Jones Y, Rice P, Derwent S, Odondi L (2010). The use of fixed appliances in the UK: a survey of specialist orthodontists. J Orthod.

[4] Keim RG, Vogels DS, Vogels PB (2020). 2020 JCO study of orthodontic diagnosis and treatment procedures Part1 results and trends. J Clin Orthod.

[5] Streva AM, Cotrim-Ferreira FA, Garib DG, Carvalho PE (2011). Are torque values of preadjusted brackets precise?. J Appl Oral Sci.

[6] Anjos A, Pompeo DD, Enricone dos Anjos GJ, Stefan Oliveira GM, Rosário HD (2015). Assessment of torque angle of brackets from different brands. Brazilian J Oral Sci.

[7] Mendonca MR, Verri AC, Fabre AF, Cuoghi OA (2014). Analysis of mesiodistal angulations of preadjusted brackets. Braz Oral Res.

[8] Dalstra M, Eriksen H, Bergamini C, Melsen B (2015). Actual versus theoretical torsional play in conventional and self-ligating bracket systems. J Orthod.

[9] Joch A, Pichelmayer M, Weiland F (2010). Bracket slot and archwire dimensions: manufacturing precision and third order clearance. J Orthod.

[10] Cash AC, Good SA, Curtis RV, McDonald F. An evaluation of slot size in orthodontic brackets - Are Standards as expected? Angle Orthod. 2004. 10.1043/0003-3219(2004)074<0450:AEOSSI>2.0.CO;2 15387021

[11] Sernetz F (2005). Standardization of orthodontic products–does it make sense. J Orofac Orthop.

[12] Brown P, Wagner W, Choi H (2015). Orthodontic bracket slot dimensions as measured from entire bracket series. Angle Orthod.

[13] Lefebvre C, Saadaoui H, Olive JM, Renaudin S, Jordana F (2019). Variability of slot size in orthodontic brackets. Clin Exp Dent Res.

[14] Supriadi S, Sitanggang TW, Bambang Irawan S, Suharno B, Kiswanto G, Prasetyadi T (2015). Orthodontic bracket fabrication using the investment casting process. Int J Technol.

[15] Park JS, Song IT, Bae JH, Gil SM, Kang KH (2020). Comparison of slot sizes and parallelism of metal brackets manufactured through metal injection molding and computerized numerical control. Korean J Orthod.

[16] Amon CH, Beuth JL, Weiss LE, Merz R, Prinz FB (1998). Shape deposition manufacturing with microcasting: Processing, thermal and mechanical issues. J Manuf Sci Eng Trans ASME.

[17] Jackson CB. Accuracy and performance of a novel 3d metal printed orthodontic bracket. University of North Carolina at Chapel Hill; 2017.

[18] Eliades T, Zinelis S, Bourauel C, Eliades G (2010). Manufacturing of Orthodontic Brackets: A Review of Metallurgical Perspectives and Applications. Recent Patents Mater Sci.

[19] Major TW, Carey JP, Nobes DS, Major PW. Orthodontic Bracket Manufacturing Tolerances and Dimensional Differences between Select Self-Ligating Brackets. J Dent Biomech. 2010;1. doi:10.4061/2010/781321. 10.4061/2010/781321 PMC2958443 20981299

[20] Kusy RPWJ (1999). Assessment of second-order clearances between orthodontic archwires and bracket slots via the critical contact angle for binding. Angle Orthod.

[21] Eshan Awasthi N (2015). Evaluation and Comparison of Various Prescription Specifications and Slot Distortion of Pre-Adjusted Edgewise Brackets Manufactured By Different Companies Available in India -. Int J Curr Res Rev.

[22] Arreghini A, Lombardo L, Mollica F, Siciliani G (2014). Torque expression capacity of 0.018 and 0.022 bracket slots by changing archwire material and cross section. Prog Orthod.

[23] Tepedino M, Paiella G, Iancu Potrubacz M, Monaco A, Gatto R, Chimenti C (2020). Dimensional variability of orthodontic slots and archwires: an analysis of torque expression and clinical implications. Prog Orthod.

[24] Schmidli H, Grize Y-L (1997). QUANTIFICATION OF BATCH HOMOGENEITY. Qual Eng.

[25] Lee Y, Lee DY, Kim YJ (2016). Dimensional accuracy of ceramic self-ligating brackets and estimates of theoretical torsional play. Angle Orthod.

[26] Archambault A, Lacoursiere R, Badawi H, Major PW, Carey J, Flores-Mir C (2010). Torque expression in stainless steel orthodontic brackets. Angle Orthod.

[27] Siatkowski RE. Loss of anterior torque control due to variations in bracket slot and archwire dimensions. J Clin Orthod. 1999;33:508–10. http://www.ncbi.nlm.nih.gov/pubmed/10895655. 10895655

[28] Gioka C, Eliades T (2004). Materials-induced variation in the torque expression of preadjusted appliances. Am J Orthod Dentofac Orthop.

[29] Kancab Díaz R, del C, Ruiz Díaz, Ruiz Botello R, Padilla Olvera G. S. Tolerance in a 0.022” x 0.025” bracket slot from three commercial brands used in the Department of Orthodontics of the National Autonomous University of Mexico. Rev Mex Ortod. 2014;2:e188–91.

